# Experimental evidence of physician social preferences

**DOI:** 10.1073/pnas.2112726119

**Published:** 2022-07-06

**Authors:** Jing Li, Lawrence P. Casalino, Raymond Fisman, Shachar Kariv, Daniel Markovits

**Affiliations:** ^a^The Comparative Health Outcomes, Policy, and Economics (CHOICE) Institute, Department of Pharmacy, University of Washington, Seattle, WA 98195;; ^b^Department of Population Health Sciences, Joan & Sanford I. Weill Medical College of Cornell University, New York, NY 10065;; ^c^Department of Economics, Boston University, Boston, MA 02215;; ^d^Department of Economics, University of California, Berkeley, CA 94720;; ^e^Yale Law School, Yale University, New Haven, CT 06511

**Keywords:** physicians, social preferences, altruism, equality, efficiency

## Abstract

Physicians’ professional ethics require that they put patients’ interests ahead of their own and that they should allocate limited medical resources efficiently. Understanding physicians’ extent of adherence to these principles requires understanding the social preferences that lie behind them. These social preferences may be divided into two qualitatively different trade-offs: the trade-off between self and other (altruism) and the trade-off between reducing differences in payoffs (equality) and increasing total payoffs (efficiency). We experimentally measure social preferences among a nationwide sample of practicing physicians in the United States. Our design allows us to distinguish empirically between altruism and equality–efficiency orientation and to accurately measure both trade-offs at the level of the individual subject. We further compare the experimentally measured social preferences of physicians with those of a representative sample of Americans, an “elite” subsample of Americans, and a nationwide sample of medical students. We find that physicians’ altruism stands out. Although most physicians place a greater weight on self than on other, the share of physicians who place a greater weight on other than on self is twice as large as for all other samples—32% as compared with 15 to 17%. Subjects in the general population are the closest to physicians in terms of altruism. The higher altruism among physicians compared with the other samples cannot be explained by income or age differences. By contrast, physicians’ preferences regarding equality–efficiency orientation are not meaningfully different from those of the general sample and elite subsample and are less efficiency oriented than medical students.

## Significance

Physicians routinely face trade-offs among their own interests, the interests of their patients, and society’s interest in preserving medical resources. To manage these trade-offs, society relies on both traditional professional ethics and bureaucratic monitoring and control. Our results—that physicians are twice as likely to be altruistic as all other samples but indistinguishable from the general population in terms of equality–efficiency orientation—suggest that professional norms can meaningfully contribute to physicians putting patients first and highlight the importance of nurturing these norms of physician professionalism. However, our findings also suggest that policymakers may not rely on physician professionalism to ensure an efficient allocation of medical resources.

In a classic article, Kenneth Arrow (1) argued that asymmetric information pervades the health-care market. Patients rely on physicians’ expert knowledge in planning their medical care. Health insurers and government agencies (Medicare and Medicaid) largely rely on physicians to decide which treatments are appropriate for their patients. This deference to physicians’ authority may be justified given their superior expertise and informational advantages (2). However, the dual role of recommending and providing treatments creates opportunities for physicians to place their interests ahead of their patients’ interests, for example by recommending profitable tests and treatments that offer little or no health benefits. A second risk is more subtle. Physicians must trade off their individual patients’ interests in getting care, even if the benefit is likely to be small, against society’s interest in allocating limited medical resources efficiently, in order to generate the greatest benefits for the overall health of a population.

The norms of physician professionalism—including, in particular, the patient-centered norms that constitute physicians’ traditional professional ethic—are intended to address the risk of selfishness. Arrow argues that due to information asymmetry, the principle of“buyer beware” that governs ordinary consumer markets should be replaced, in health care, by the physicians’ professional responsibility to put patients’ interests ahead of their own (1). Physician leaders publicly promote the importance of professionalism, while exhorting physicians to act altruistically. For example, the editors of the *New England Journal of Medicine* have asserted that “medicine is one of the few spheres of human activity in which the purposes are unambiguously altruistic” (3), while the American Board of Internal Medicine similarly asserts that “altruism is the essence of professionalism … the best interest of patients, not self-interest, is the rule” (4). On the other hand, empirical studies have suggested that, at least in some situations, some physicians create “supplier-induced demand,” which influences a patient’s demand for care “against the physician’s interpretation of the best interest of the patient” (5), contributing to skepticism about whether physicians do in fact behave altruistically. [Such skepticism is not limited to medicine. Legal ethics, for example, has long sought to control lawyers’ abuse of discretion through professional norms of client loyalty and care (6). But skeptics have cast these norms as self-serving, and the law governing lawyers increasingly subjects them to elaborate institutionalized mechanisms of bureaucratic control.]

While the effects of professional norms on physician behavior are difficult to measure directly, a clearer understanding of physicians’ social preferences can help to illuminate whether professional norms and physicians’ individual preferences are oppositional or aligned. Our study therefore helps to evaluate the likely effectiveness of both professional norms and the turn to bureaucracy. While altruism and related professional norms are important in many other professions (7), the distinct characteristics of the market for medical care, namely information asymmetry and uncertainty in the relationship between medical treatments and patient outcomes (1), render it especially critical to study these issues among physicians.

Health care systems in the US and elsewhere address the second risk—concerning efficiency—in more complex ways. Although professional ethics give physicians a responsibility to conserve scarce medical resources (8), the norm that directs individual physicians to put their patients first may render a norm-based approach inadequate to the problem of efficiency (9). Health insurers therefore use bureaucratic mechanisms and financial incentives to manage the information asymmetry between a physician who knows the specific patient’s situation and the insurer which does not (9).*

We deploy an incentivized economic experiment to investigate both altruism (the trade-off between self and other) and equality–efficiency orientation (the trade-off between reducing self–other differences in payouts and increasing payout totals) in practicing US physicians, and we compare our results with analogous experiments that measure parallel behaviors in other populations. A vast literature considers social preferences, and laboratory experiments have been very fruitful in both establishing the empirical reliability of such preferences and directing theoretical attention to them. [We will not attempt to review the enormous body of work in behavioral and experimental economics on social preferences. Camerer (10) provides a comprehensive discussion, if now somewhat dated, of the vast body of experimental and theoretical research in economics focusing on dictator, ultimatum, and trust games. Engel (11) provides the most comprehensive meta-study of dictator games.] After presenting our results, we relate them to the results from prior work that are particularly relevant to our study (*Discussion*). We note that the social preferences of physicians and professionals more generally remain relatively understudied, and our discussion of the relationship between our study and prior work explains the specific contributions that we make.

Our sample consists of 284 physicians from 36 medical groups around the United States, including physicians in primary care (internal medicine and family medicine) and cardiology, and physicians in private practices and employed by hospitals. Our experiment gives subjects broad discretion to implement their preferences, free from bureaucratic control or even surveillance. Our results therefore inform the question whether norms are likely to affect physician choices along both dimensions of behavior. Our study measures altruism in a large multisite sample of practicing physicians and measures both dimensions of social preferences.

Our experiment asked subjects to make trade-offs between their own self-interest and the interest of an anonymous other and, at the same time, between equality and efficiency. These two aspects of social preferences often operate together, but they remain conceptually distinct. [Social preferences can be weighted toward equality (reducing differences in payoffs) or weighted toward efficiency (increasing total payoffs) and range from pure utilitarian to maxmin or Rawlsianism. As the dispute between Harsanyi (12, 13) and Rawls (14) shows, fair-minded people (who are all perfectly impartial between self and other) can disagree about how to trade off equality and efficiency. The work of Harsanyi and Rawls, and of the many others who have followed them, has had broad-reaching influence across many disciplines, including philosophy, economics, and law.] To capture both of these features in our experiment, we employ a modified dictator game (15–17) in which we ask physicians to allocate real money between themselves and an anonymous other drawn from a broadly representative sample of the US population. Our experiment presents subjects with allocation decisions in which the “price of giving” varies across decision problems—sometimes the subject may need to sacrifice more than a token (the experimental currency)—to give a single token to other (the recipient); in other decisions, it may cost only a fraction of a token. These decisions are made through an intuitive “point-and-click” graphical interface in which the choices are represented as a budget line where each point represents a possible allocation. The slope of the line captures the price of giving tokens to other.^†^

Intuitively, this method allowed us to collect a rich dataset capable of measuring both altruism and equality–efficiency orientation at the level of the individual subject. [The importance of studying individual heterogeneity in social preferences is emphasized by Andreoni and Miller (17). Because of this heterogeneity, it is necessary to investigate behavior at an individual level. Our experimental design allows subjects to make numerous choices over a wide range of budget lines, and this yields a rich dataset that is well-suited to analysis at the individual level. It is clearly advantageous to estimate individual-level parameters and then generate individual-level distributions of the estimations rather than to pool data and then estimate population-level parameters.] The degree of altruism is reflected in the amount subjects give on average, whereas equality–efficiency orientation is captured by how subjects respond to the price of giving. Increasing the fraction of the budget spent on other as the price of giving increases indicates social preferences weighted toward equality (reducing the difference in payoffs between self and other), whereas decreasing it when the price of giving increases indicates social preferences weighted toward efficiency (increasing the total payoffs to self and other). We rely on techniques developed in our prior work (15, 16, 18) to evaluate the consistency of physicians’ choices (i.e., whether they reflect a complete and transitive preference ordering) and to explore the structure of the social utility functions that rationalize the observed data.

We further compare physicians’ preferences with preferences previously measured in three other populations using equivalent experiments: 1) a broadly representative sample of US adults (18), 2) an “elite” subsample of those who hold a graduate degree and have an annual household income over $100,000 (15, 18), and 3) a sample of medical students from nine schools around the United States (19, 20). The social preferences of these populations provide important benchmarks against which physicians’ social preferences can be assessed; furthermore, the comparison with medical students may shed light on whether physicians’ distinctive social preferences reflect a “selection effect” based on who enters medicine or a “treatment effect” of practicing medicine.^‡^

We begin our analysis of the experimental data by using classical revealed preference theory (21–23) to test whether subjects’ choices are consistent with the essence of all traditional models of economic decision-making—utility maximization.^§^ Our physician subjects exhibit a remarkably high degree of consistency when compared with other populations, including medical students and also students from Yale Law School (YLS), the population that had exhibited the highest degree of consistency in prior experiments (15). [In our subsequent analysis, we do not draw detailed comparisons between our physician sample and the sample YLS students (15). The experimental design in Fisman et al. (15) differs from the current one in that the YLS student subjects were asked to allocate money between themselves and another student, rather than an individual drawn from a sample broadly representative of the US adults.] This result reveals that our physician subjects are highly adept at implementing a consistent, well-behaved social preference ordering. This makes it natural to estimate—at the level of the individual subject—the substantive social preferences that physicians display.

We then estimate social preferences at the level of the individual physician using a constant elasticity of substitution (CES) utility function commonly employed by economists in demand analysis. The CES functional form is appealing because the degree of altruism and equality–efficiency orientation are each independently represented in a precise and transparent manner through its two parameters, which we estimate separately for each subject (further details on the CES specification and estimation are provided in *Empirical Framework*).

We find that physicians are more altruistic than any other population, while physicians’ preferences concerning the trade-off between equality and efficiency are almost indistinguishable graphically from the preferences of the American Life Panel (ALP) elites and also the broader ALP sample. These findings on physicians’ distinctive social preferences have direct and concrete implications for professionalism, incentives, and bureaucratic rules directed at physicians. Insofar as physicians are altruistic, they may be more likely to live up to the professional ideal of putting patients’ interests ahead of their own. At the same time, altruism as captured in our experiment is far from ubiquitous, even among physicians and, furthermore, physicians’ efficiency orientation is indistinguishable from than that of the general population. Taken together, our findings suggest that the ideal of physician professionalism—putting the patient first—is not merely a self-serving myth but that other mechanisms may be required to support the quality of medical care and to promote efficient allocation of medical resources.

## The Subjects

### The Physician Subject Pool.

The subjects in our experiment (recruited as described in *Recruitment*) are primary care physicians (internal medicine and family practice) and cardiologists. These specialties represent a wide income range which itself may be associated with differences in social preferences. Cardiology is one of the highest-paying physician specialties in the United States, with an average annual income of $430,000 in 2019 (24). Internal medicine and family medicine are primary care specialties at the lower end of the physician income distribution, earning on average $243,000 and $231,000, respectively, in 2019 (24).

The ages of our physician subjects range from under 30 y to over 60 y; 39% are female, with a much higher fraction of females in primary care (50%) compared with cardiology (18%). A quarter of physicians worked in private practices and the rest in hospitals (including academic medical centers). Practice sizes ranged from 8 to 1,600 physicians; 41% of the physicians in our sample practice in the Northeast (census region I), 24% in the Midwest (census region II), 17% in the South (census region III), and 18% in the West (census region IV).

Our physician subject pool is thus heterogeneous in terms of age, practice type, practice size, and location of practice. Finally, except for gender, the demographic differences between our physician subjects from primary care and cardiology are relatively minor. Our final sample includes 284 physicians (131 in internal medicine, 57 in family medicine, and 96 in cardiology) from 36 medical groups, after excluding 7 groups with a single participant from each. Table 1 summarizes the characteristics of the physician sample.

**Table 1.** Summary statistics of the individual characteristics of the physician sample

	Primary care	Cardiology	Total
Female	0.495	0.177	0.387
Age, y			
≤39	0.346	0.208	0.299
40–49	0.261	0.458	0.327
50–59	0.250	0.156	0.218
≥60	0.144	0.177	0.155
Region			
Northeast	0.468	0.292	0.408
Midwest	0.239	0.240	0.239
South	0.074	0.354	0.169
West	0.218	0.115	0.183
Practice type			
Hospital	0.755	0.750	0.754
Private	0.245	0.250	0.246
Practice size			
≤35	0.176	0.115	0.155
36–100	0.356	0.271	0.327
101–350	0.207	0.062	0.158
350–1,600	0.261	0.552	0.359
Observations	188	96	284

Fraction of subjects. Primary care includes internal medicine and family medicine. Regions according to the US Census Bureau census regions: Northeast (census region I), Midwest (census region II), South (census region III), and West (census region IV).

### Comparison Subject Pools.

To compare the social preferences of physicians with those of the general US population, we drew data from an equivalent experiment with subjects from the ALP reported in Fisman et al. (18). The demographic and socioeconomic characteristics of ALP respondents resemble the broader US population.^¶^ Our ALP sample consists of 993 subjects and closely matches the general population in terms of age, place of residence, education, race, and income. [Fisman et al. (18) compared the subjects in the experiment with both the entire ALP sample and to the American Community Survey (ACS) conducted by the US Census and representative of the US population. The subsample of ALP subjects in the experiment is consistent with the entire ALP sample and with the general population in the ACS.]

We are specifically interested in assessing whether social preferences measured among physicians reflect the distinct attributes of the medical profession or simply preferences of a more elite class compared with the general population. We therefore used data from an elite ALP sample (15) that overlaps with the general ALP sample but is not a subset of the latter (18). Following our previous work (15), we define an ALP respondent as elite if they 1) have a graduate degree, 2) are employed, and 3) have annual household income of at least $100,000. The ALP elite sample consists of 82 subjects, with average household income of approximately $127,600. [The ALP elite sample is smaller than the other samples, but the number of ALP elite subjects is still higher than is usual in the literature, and the experiments provide us with a rich dataset consisting of enough individual decisions over a wide range of budget lines to provide a powerful test of the social preferences of elites. Our ALP elite sample is also larger than the elite sample of 54 subjects in Fisman et al. (15).]

To explain the distinctiveness of physicians’ social preferences, we add data on a sample of 503 medical students who completed an equivalent experiment (19, 20). The students were recruited across all 4 y of study in nine medical schools around the United States. Analyzing medical students alongside physicians allows us to compare social preferences among individuals at different stages of the same profession and provides evidence concerning the distinct effects of selection (into medicine) and treatment (practicing medicine) on physicians’ social preferences.

Among physician subjects, 39% are female, compared with 58% in the general ALP sample, 55% among ALP elites, and 46% in the medical student sample. Approximately 63% of the physician sample was younger than 50 y; 30% were younger than 40 y. By comparison, 48% of the general ALP sample were younger than 50 y; 79% of ALP elites were younger than 50 y, with about 64% younger than 40 y because of oversampling of those aged 40 y and below. Almost all medical student subjects were younger than 40 y. All our results below are robust to the inclusion of controls for gender and age (when appropriate). Table 2 compares the characteristics of the physician sample with those of the other samples: ALP, ALP elites, and medical students.

**Table 2.** A comparison of the individual characteristics of the physician sample and the three other samples

	Physicians	General ALP	ALP elites	Medical students
Female	0.387	0.584	0.549	0.455
Age, y				
≤39	0.299	0.294	0.637	0.998
40–49	0.327	0.189	0.154	0.002
50–59	0.218	0.264	0.132	0
≥60	0.155	0.253	0.077	0
Region				
Northeast	0.408	0.177	0.293	0.131
Midwest	0.239	0.202	0.207	0.235
South	0.169	0.354	0.281	0.479
West	0.183	0.267	0.220	0.155
Observations	284	993	82	503

Fraction of subjects. Regions according to the US Census Bureau census regions: Northeast (census region I), Midwest (census region II), South (census region III), and West (census region IV).

## Methods

### Recruitment.

We recruited physicians by approaching (via email) leaders of medical groups that include the relevant specialties and requesting that they make their members aware of an opportunity to participate in a study of physician decision-making. There is no generally accepted national database of medical groups, so we identified groups in three ways: 1) via group leaders known to one of the authors (L.P.C.), who has studied medical groups in the United States since 1994, 2) via group leaders referred by one of the author’s (L.P.C.) contacts, and 3) via web searches. None of the authors has a close relationship with any medical group in any of these three categories.

We approached leaders of primary care groups (internal medicine and/or family practice), of cardiology groups, and of multispecialty groups that included primary care physicians and/or cardiologists. Within these specialties, our objective was to include groups that varied by size and geographic region of the United States. Of the 87 group leaders who were contacted via email between October 2018 and November 2019, 43 groups participated.

A natural limitation of our physician sample is that it is not a random sample of all practicing physicians (or physician groups) in the United States and thus can be subject to selection concerns. However, as we discuss below, we found no significant differences in altruism or efficiency orientation between medical groups contacted because they were known to one of the authors, those that were suggested by contacts of the author’s (L.P.C.), and those that were found by web searches. There was also no significant difference in altruism or efficiency orientation by group size. While we cannot rule out selection bias, these results suggest that selection at the group level is unlikely to be important in driving our results. [More generally, those who select into laboratory experiments have been shown to be slightly less altruistic than those who do not (25), again arguing broadly against selection bias as a first-order concern.]

### The Experiment.

Our proposed analysis and new experiments draw on our prior work (15, 16, 18), which was motivated by the need to provide a better positive account of social preferences. To provide that account, we need a choice environment that is rich enough to allow a general characterization of the patterns of individual behavior. In addition, characterizing behavior at the level of the individual subject requires generating many observations per subject over a wide range of choice sets. Fisman et al. (16) developed a graphical interface for exactly this purpose. [Our experimental method has been applied to many types of individual choice problems involving attitudes toward risk, time, and inequality. Fisman et al. (15, 18), Li (19), and Li et al. (20) build on the work in Fisman et al. (16) to study social preferences with various, diverse samples. Ahn et al. (26) extended the work in Choi et al. (27) on risk (known probability) to settings with ambiguity (unknown probability). Building on the experimental methodology and utilizing a nationally representative panel, Choi et al. (28) relate findings on individual-level behaviors from the experimental data with subjects’ economic and sociodemographic characteristics. Because all experimental designs share the same graphical interface, we are building on expertise we have acquired in previous work.] With the interface, subjects see on a computer screen a geometrical representation of a standard consumer decision problem (selection of a bundle from a standard budget set) and choose allocations through a simple point and click. [It is possible that presenting choice problems graphically biases choice behavior in some particular way, but there is no evidence that this is the case—average behavior elicited graphically is quite consistent with behavior elicited by other means.]

The computer interface and experimental protocols, developed for our research, have been integrated with the Understanding America Study (UAS) and ALP online survey instruments. Conducting the experiments online ensured anonymity and effective isolation of subjects in order to minimize any interpersonal influences that might stimulate other-regarding behavior. The web-based experiment conducted with physician subjects is identical to the previous experiments with ALP and medical student subjects. [The experiments with physicians were conducted several years after the experiments with medical students and with the ALP. We cannot rule out that differences in social preferences across samples could be related to temporal differences in preferences. Fisman et al. (29) examine the intertemporal stability of social preferences across several years in an identical experiment to ours with the ALP and find that the individual-level CES estimates of altruism (*α*) and equality–efficiency orientation (*ρ*) based on the choices in 2013 are highly predictive of those estimates based on choices 3 y later in 2016.] In the experimental task, the choices made by self (the subject) have consequences for her own payoff and the payoffs of an unknown other—in all experiments an anonymous respondent from a representative sample of the US (adult) population. All experimental subjects received the same information on the sample from which respondents are drawn, which includes a substantial amount of demographic, socioeconomic, and geographic diversity.

Throughout, we denote persons self and other by s  and o, respectively, and the associated monetary payoffs by $\pi_{s}$ and $\pi_{o}$. Since a great deal of classical decision theory is built on the assumption of a linear budget constraint, we study a modified dictator game in which self must allocate an endowment across $\pi_{s}$ and $\pi_{o}$ at prices $p_{s}\;$ and $p_{o}$. Without essential loss of generality, assume the endowment is normalized to 1. The set of budget lines is then

$$p_{s}\pi_{s}+p_{o}\pi_{o}=1.$$

As we explain below, varying the relative price of giving $p_{s}/p_{o}$ across decisions allows us separately to identify subjects’ altruism (the relative weight on $\pi_{s}$ versus $\pi_{o}$) and equality–efficiency orientation (the relative weight on decreasing the difference between $\pi_{s}$ and $\pi_{o}$ versus increasing the sum of $\pi_{s}$ and $\pi_{o}$). [In a standard split-the-dollar dictator experiment, first introduced by Forsythe et al. (30), self divides the endowment between self and other such that $\pi_{s}+\pi_{o}=1$. One respect in which this framework is restrictive is that the set of feasible payoff pairs is always the budget line with a slope $p_{s}/p_{o}=-1$, so that the problem faced by self is simply allocating a fixed total income between self and other, making it impossible to identify equality–efficiency orientation which requires a variation is the price of giving $p_{s}/p_{o}$.]

Each experimental subject faced 50 independent decision rounds. Each of the 50 decision rounds in the experiment began with the computer’s selecting a budget line at random. The budget lines selected for each subject in his/her decision problems were independent of each other and of the budget lines selected for other subjects in their decision problems. Subjects saw the budget lines on a computer screen and chose allocations through a simple point-and-click interface. A subject’s chosen allocation $\left(\pi_{s},\pi_{o}\right)$ determined the payoffs from a particular decision: self received $\pi_{s}$ and other (a randomly chosen anonymous respondent from the general population not sampled for the experiment) received $\pi_{o}$.^#^

The experiment provides us with a very rich dataset. Most importantly, the broad range of budget sets provides a serious test of the ability of classical theory to interpret the data. First, the graphical interface allows us to test a wider range of budget lines than can be tested using other experimental questionnaire methods. Second, our subject-level dataset makes it possible to study heterogeneity, which by its nature requires behavior to be examined at an individual level.^ǁ^ We refer the interested reader to Fisman et al. (16, 18) for an extended description of the experimental design and procedures.

At the end of the experiment, the computer selected one decision at random for each subject, and self and other were paid the amount they earned in that round. The round selected depended solely on chance. The maximum possible payoff to each physician subject was $250, with an average payoff of about $156 if the subject always gave nothing to other. In comparison, the maximum possible payoff (resp. average payoff) was $50 ($31) in the experiments with the ALP subjects and $25 (resp. $16) in the experiments with medical students. The payoff scale in each experiment was chosen to make the average payoff roughly match the average hourly market wage of that experiment’s subject pool (the residency wage in the case of medical students).**

Following the experiment, the physician subjects were asked to complete a brief survey questionnaire, which elicited their sociodemographic information as well as information on the nature of their practices. The payment from the experiment was contingent on completing the survey.

### Empirical Framework.

We begin by measuring the extent to which subjects’ behavior is consistent with utility maximization. Following classical revealed preference theory, we employ the Generalized Axiom of Revealed Preference (GARP) to test whether individual subjects’ choices in our experiment can be rationalized by a well-behaved (continuous, increasing, and concave) utility function $u_{s}\left(\pi_{s},\pi_{o}\right)$ that captures social preferences. To assess how nearly the data comply with GARP, we calculate Afriat’s Critical Cost Efficiency Index (CCEI). The CCEI is bounded between 0 and 1; the closer it is to 1, the smaller the perturbation of budget sets required to remove all GARP violations and thus the closer the data are to being perfectly consistent with economic rationality. We provide more details on GARP and CCEI in *Materials and Methods*.

After determining that subjects’ choices are approximately consistent with rationality, we further assume that the underlying utility function $u_{s}\left(\pi_{s},\pi_{o}\right)\;$ is a member of the CES family commonly employed in demand analysis and found by prior experimental work to be appropriate for capturing social preferences. For our purposes, the advantages of the CES formulation are therefore flexibility, tractability, and straightforward interpretation. The CES utility function is given by

us=[απsρ+(1−α)πoρ]1ρ.

The parameters *α* and *ρ* capture distinct elements of individuals’ distributional preferences. The parameter α∈[0,1] measures altruism. α=1/2 indicates fair-mindedness (impartial treatment of self vis-à-vis other), whereas α=1  indicates pure selfishness and α=0 indicates pure selflessness. [Ellis and McGuire (31) and others use α to denote the rate at which the physician is willing to trade off one dollar of hospital profit for one dollar of patient benefit, which is related but conceptually distinct from our use of α as defined in the CES utility function. We note this distinction while maintaining the notations above to keep them consistent with prior literature using the same methodology.] The parameter ρ≤1  measures equality–efficiency orientation—the willingness to trade off equality and efficiency in response to price changes—and ρ/(ρ−1) is (constant) elasticity of (social) substitution between self and other. As ρ→0, the CES utility function approaches the Cobb–Douglas utility function, $\pi_{s}^{\alpha }\pi_{o}^{1-\alpha }$, so the expenditures of tokens to self $p_{s}\pi_{s}\;$ and other $p_{o}\pi_{o}\;$ are constant and equal to α and 1−α, respectively, for any relative price of giving $p_{s}/p_{o}$. Any ρ>0 (resp. ρ<0) indicates social preferences weighted toward efficiency (resp. equality) because $p_{s}\pi_{o}$ decreases (resp. increases) when the relative price of giving $p_{s}/p_{o}$ decreases. We provide further details on the CES formulation and the individual-level econometric estimation of *α* and *ρ* in *Materials and Methods*.

### A Note on External Validity.

Our earlier work also bolsters the external validity of our findings, which Levitt and List (32) point out is a critical concern for experimental studies of social preferences. Indeed, the relevance of experimental studies in economics (and other social sciences) rests on the assumption that behavior in the laboratory is correlated in a reasonable way (although presumably imperfectly) with behavior outside the laboratory. Li (19) shows that the experimental measure of altruism based on our design strongly predicts medical students’ self-reported specialty and career decisions: Conditioning on extensive covariates, those with lower altruism are more likely to choose high-income specialties and less likely to report planning on practicing medicine in underserved areas.

Fisman et al. (18) demonstrate the predictive validity of our experimental measures by documenting a relationship between social preferences and political decisions in the ALP—equality-focused subjects are more likely to have voted for Barack Obama in 2012 and to be affiliated with the Democratic Party. Fisman et al. (15) show that more efficiency-focused behavior in the laboratory was associated with a greater likelihood of choosing private sector employment after graduation, whereas more equality-focused behavior was associated with a greater likelihood of choosing nonprofit sector employment. Choi et al. (28) present further instances in which parameters measured in the laboratory correlate with corresponding behaviors in the world. [Choi et al. (28) chose to investigate wealth because its accumulation is determined by countless individual decisions, made over time in many different environments, and involving a host of different trade-offs concerning risk, time, and personal and social consumption. Several studies document large wealth differentials among households with similar lifetime income. Furthermore, these wealth differentials cannot be fully explained either by standard observables, such as family structure or income volatility, or by preference-based measures, such as risk tolerance or intertemporal substitution. Predicting wealth differentials thus provides a particularly strong test of external validity.]

## Results

### Overview.

We seek first to test the rationality (completeness and transitivity) of physicians’ social preferences and then to give these preferences a substantive parametric characterization using the CES family of (social) utility functions. To do so we calculate our physician subjects’ CCEI scores and estimate the CES parameters *α* (to measure altruism) and *ρ* (to measure equality–efficiency orientation) at the level of the individual subject. We also compare physicians—with respect to CCEI, *α*, and *ρ*—with the other populations that we have investigated.

**Fig. 1.**
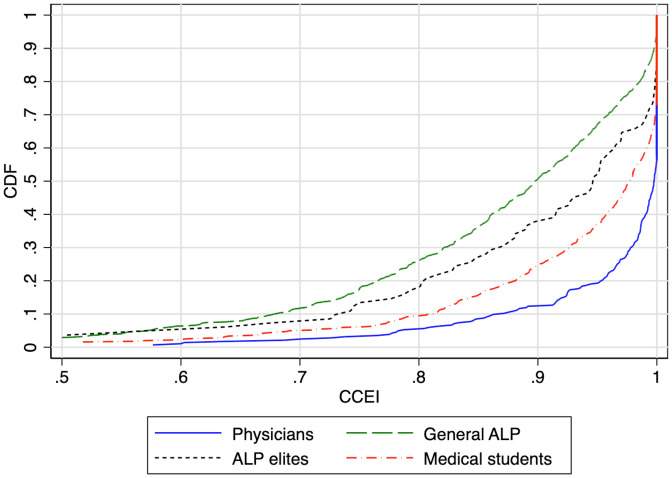
Cumulative distributions of the CCEI in the physician sample and the three other samples.

We begin in Fig. 1 with the cumulative distribution functions (CDFs) of the CCEI scores for physicians as well as other subject groups, as a measure of each sample’s rationality. The mean CCEI in the physician sample is 0.96, and the median is 0.998, indicating that the overwhelming majority of physicians are perfectly or almost perfectly rational. While the rationality of physicians is remarkably high when compared with the two ALP samples, overall we find that across all groups most subjects exhibit GARP violations that are minor enough to ignore for the purposes of recovering social preferences by constructing appropriate utility functions—even for the ALP sample the mean CCEI score is 0.86 and the median is 0.90.^††^ We interpret the CCEI scores as confirmation that subject choices are generally consistent with utility maximization. We can therefore move to recovering underlying social preferences by estimating CES functions at the individual level.

**Fig. 2.**
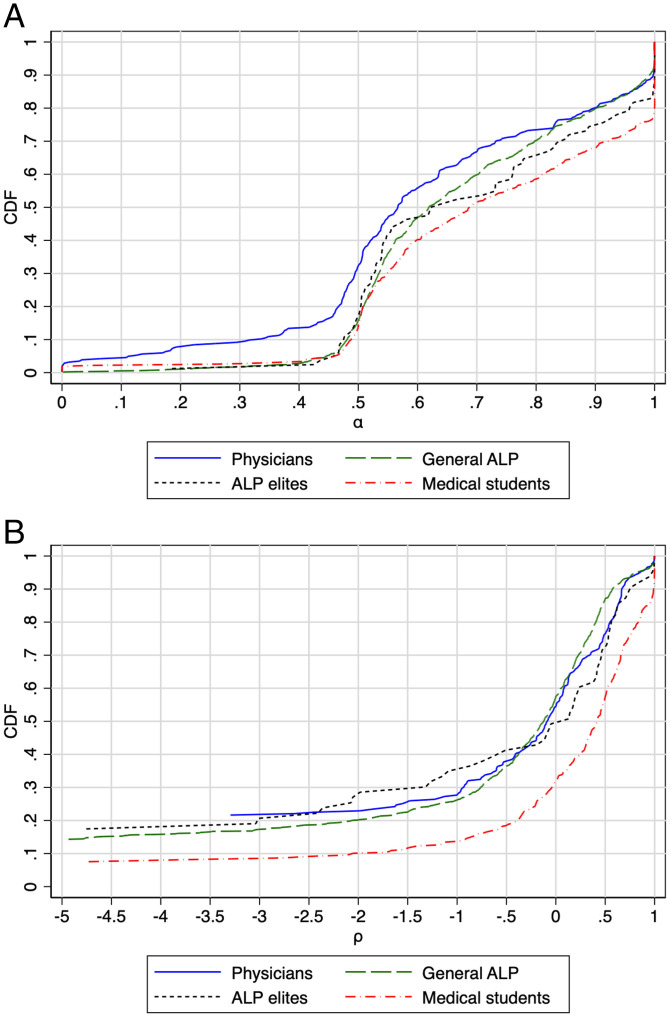
Cumulative distributions of the (*A*) estimated altruism (α) and (*B*) equality–efficiency orientation (ρ) in the physician sample and the three other samples.

In Fig. 2, we provide CDFs based on the individual-level CES estimates of altruism (α) and equality–efficiency orientation (ρ) to compare the social preferences of physicians with those of other samples.^‡‡^ Physicians are more altruistic than all three comparison samples (Fig. 2*A*) as the CDF of the estimated altruism parameter (α) of the physician sample is skewed to the left. Of particular note, physicians are twice as likely to put equal or greater weight on other relative to self (α≤1/2)—32% as compared with 15 to 17% for the ALP general population, ALP elites, and medical students. By contrast, physicians’ preferences concerning the trade-off between equality and efficiency (Fig. 2*B*) are almost indistinguishable graphically from the preferences of the ALP elites and also the broader ALP sample. The CDF of the estimated equality–efficiency orientation (ρ)  parameter of the physician sample is only visibly to the left of the CDF of the medical student sample, indicating that physicians are less efficiency-oriented than the medical students.^§§^

Table 3 reports the results of normalized rank transformation regressions, which assess the statistical significance of the differences between the social preferences of physicians and each of the other samples and measure the magnitudes of these differences. Specifically, a rank transformation regression transforms the combined data from two populations into an overall ranking, which is then normalized to have mean 0 and SD 1. In each case, the coefficient on the indicator variable “Physician” measures the effect of being a physician on a subject’s place, in terms of SDs, in the composite distribution. This approach has the merit of making the coefficients comparable across the three variables of interest—the CCEI and the CES parameters *α* and *ρ*—which have very different underlying distributions. Consistent with the CDFs in Figs. 1 and 2, physicians are significantly more rational (columns 1 through 3) and more altruistic (columns 4 through 6) than any other sample but very similar to both the general and elite ALP (columns 7 and 8) samples. *SI Appendix*, Table A1 reports regressions results without rank transformation.^¶¶^ We discuss these results in more detail in the following sections.

**Table 3.** Rationality (CCEI), altruism (*α*), and equality–efficiency orientation (*ρ*) in the physician sample and the three other samples

	CCEI			*α*			*ρ*		
	(1) vs. General ALP	(2) vs. ALP elites	(3) vs. MS	(4) vs. General ALP	(5) vs. ALP elites	(6) vs. MS	(7) vs. General ALP	(8) vs. ALP elites	(9) vs. MS
Physicians	0.95****	0.70****	0.33****	−0.29****	−0.35***	−0.49****	−0.00	−0.04	−0.66****
	(0.06)	(0.13)	(0.08)	(0.08)	(0.12)	(0.08)	(0.08)	(0.15)	(0.08)
Female	Yes	Yes	Yes	Yes	Yes	Yes	Yes	Yes	Yes
Age	Yes	Yes	No	Yes	Yes	No	Yes	Yes	No
Census region	Yes	Yes	Yes	Yes	Yes	Yes	Yes	Yes	Yes
*N*	1,277	366	787	1,277	366	787	1,069	294	588
R-squared	0.19	0.13	0.07	0.03	0.04	0.05	0.02	0.04	0.12

The coefficient on the indicator variable Physician measures the effect of being a physician on a subject‘s place in the composite distribution of CCEI (columns 1–3), *α* (columns 4–6), and *ρ* (columns 7–9). The *ρ* parameter of purely selfless (*α* = 0) and purely selfish (*α* = 1) subjects, who always give nothing or everything, cannot be identified. In the regressions reported in columns 7–9, we thus omit purely selfless and purely selfish using a one-sided test at the 10% level. SEs are in parentheses, bootstrapped using 500 repetitions. ***P < 0.01 and ****P < 0.001.

It is also natural to consider differences in social preferences within the sample of physicians. We distinguish between medical specialties, practice type and size, geographic divisions, and recruitment method. We consider differences in both CES parameters as well as CCEI scores and find that no discernable difference emerges along any dimension. However, the size of each subsample is relatively small, so any test for differences (or lack thereof) is underpowered. Studying the differences in social preferences among physicians is an important topic for future work, with a larger sample and potentially across a broader range of specialties. To economize on space, this analysis is provided in *SI Appendix*, Table A2.

We next compare the social preferences of physicians with those of the general population—as captured by a diverse sample of ALP subjects—and to those of other subjects with high educational attainment and incomes—as captured by the sample of elite ALP subjects. We then turn to a comparison of physicians and medical students to better understand the role of selection versus treatment.

### Physicians versus the General Population and Elites.

The first distinctive feature of physicians’ social preferences is their extremely high degree of rationality relative to both the ALP and ALP elite comparison groups. As shown in Table 3, physicians are significantly higher-ranked in their rationality—as captured by CCEI scores—relative to the ALP subjects as well as to the subset of ALP elites, and the effect size is very large. As compared with ALP subjects (column 1), physicians’ CCEIs are ranked on average 0.95 SD higher. When compared with ALP elites (column 2), physicians are ranked on average 0.70 SD higher. These results are robust to the inclusion of controls for gender and age.

We now turn to the comparison of our estimates of the individual-level CES parameters of physicians with those of the general population. This exercise reveals the other distinctive feature of physicians’ social preferences: their high level of altruism (α). This is directly observable in the raw data, in Fig. 2*A*, where the CDF of the physician sample is skewed to the left, which provides a clear graphical illustration of the extent to which the physician subjects are more altruistic than the subjects in the three other samples. Turning to our rank regression results in Table 3, we find that the differences in altruism are statistically significant and large as reflected by rank differences between physicians and the general population—physicians’ ranks are 0.29 SD lower than the ALP subjects (column 4) and 0.35 SD lower than ALP elite subjects (column 5), controlling for gender, age, and census region.

On the other hand, physicians’ equality–efficiency orientation (ρ) is not distinctive relative to the ALP general population or to the ALP elite, as can be directly observed in the raw data in the Introduction’s Fig. 1*B*. In rank regressions reported in Table 3, we confirm that the differences in ρ are extremely small and statistically insignificant, when compared with both ALP samples (columns 7 and 8). [As noted above, the transformation of the *ρ* parameter estimates to ranks has the further merit of limiting the influence of outliers in the analyses involving ρ, as its distribution has a very long left tail (lower values of −∞<ρ≤1 indicate greater equality-orientation).] The very different results for altruism (α) versus equality–efficiency orientation (ρ) highlight the fact that, although the two dimensions of social preferences often operate together, they are conceptually distinct. These results are consistent with those in *SI Appendix*, Table A1 using the raw values of each parameter as the dependent variable.

### Physicians versus Medical Students.

For the differences in rationality and altruism between physicians and the general population—as well as the ‘nondifference’ in equality–efficiency orientation—we wish to investigate whether it is driven by “selection” into medicine or the “treatment” effect of practicing medicine. We note that the comparison of physicians with the ALP elite sample suggests that the differences documented above are unlikely to be explained by the most obvious underlying differences between physicians and the broader population: education and income.

Whereas the differences in social preferences between physicians and the general population possibly capture a combination of selection and treatment, the comparison of physicians with medical students focuses on the treatment margin—by definition, medical students reflect physician behavior in the earlier parts of their careers. [The graduation rate of US medical schools is about 95%, so it is unlikely that a subgroup of medical students with particular social preferences select into becoming practicing physicians (33).] Of course, it also involves a comparison of two groups of very different ages and from distinct birth cohorts, and we therefore consider the extent to which age or cohort effects may account for various differences between physicians versus medical students at the end of this section.

First, we characterize the differences between physicians and medical students by running through the same sets of comparisons as in the preceding subsection. The normalized rank transformation regressions reported in Table 3 show that the physician subjects are, respectively, more rational, more altruistic, and much less efficiency-oriented: Physicians’ CCEIs are ranked on average 0.33 SD higher than those of medical students (column 3). The physicians’ *α* ranks are 0.49 SD lower (column 6) and their *ρ* ranks are 0.66 SD lower than those of the medical students (column 9). These results are robust to the inclusion of controls for gender and census region. The CDFs of the estimated CES parameters *α* and *ρ* in Fig. 1 and the CCEI scores in Fig. 2 reinforce these findings, as well as those in *SI Appendix*, Table A1.

In a final piece of analysis, we show that the differences in economic rationality (CCEI) and altruism (*α*) between physicians and medical students are unlikely to be driven by differences in age or income, but the large differences in equality–efficiency orientation (ρ) may plausibly be attributed to age. Our approach is as follows. We assert that there are three primary dimensions along which physicians and medical students may differ: age, income, and experience practicing medicine. If, say, age differences accounted for the gap in altruism between physicians and medical students, we would expect to observe similar differences for old versus young ALP subjects. If we observe that there is no association between age or income and our measures of social preferences in the general population, then the differences in social preferences between physicians and medical students may be attributed instead to the experience of practicing medicine (we discuss a few other possibilities below). Table 4 reports normalized rank regressions analyzing the relationship between our various outcomes of interest—the CCEI and the estimated CES parameters *α* and *ρ*—and age and income in the combined ALP samples. *SI Appendix*, Table A3 reports regressions results without rank transformation.

**Table 4.** The effect of age and income on rationally (CCEI), altruism (*α*), and equality–efficiency orientation (*ρ*) in the ALP samples

	(1) CCEI	(2) *α*	(3) *ρ*	(4) CCEI	(5) *α*	(6) *ρ*	(7) CCEI	(8) *α*	(9) *ρ*
Female	−0.24****	−0.11*	−0.15**	−0.22****	−0.09	−0.12*	−0.22****	−0.08	−0.17**
	(0.07)	(0.06)	(0.07)	(0.07)	(0.06)	(0.07)	(0.06)	(0.06)	(0.07)
Elite	0.38***	0.10	0.11						
	(0.12)	(0.12)	(0.14)						
Age, y									
31–39	−0.04	−0.02	−0.48****				−0.03	−0.05	−0.47****
	(0.11)	(0.12)	(0.11)				(0.10)	(0.11)	(0.11)
40–49	0.03	−0.06	−0.53****				0.01	−0.07	−0.54****
	(0.11)	(0.11)	(0.11)				(0.11)	(0.12)	(0.11)
50–59	0.09	−0.00	−0.57****				0.06	−0.03	−0.56****
	(0.10)	(0.10)	(0.10)				(0.10)	(0.11)	(0.10)
≥60	0.00	0.20*	−0.67****				−0.03	0.17	−0.65****
	(0.10)	(0.11)	(0.10)				(0.10)	(0.11)	(0.10)
Income									
Second quintile				0.13	0.12	−0.15	0.14	0.10	−0.15
				(0.10)	(0.10)	(0.10)	(0.09)	(0.10)	(0.10)
Third quintile				0.12	0.25***	−0.14	0.14	0.22***	−0.13
				(0.09)	(0.09)	(0.10)	(0.09)	(0.08)	(0.10)
Fourth quintile				0.23**	0.32****	−0.28***	0.23**	0.32****	−0.24**
				(0.10)	(0.09)	(0.10)	(0.10)	(0.09)	(0.10)
Fifth quintile				0.34****	0.30***	−0.04	0.34****	0.31***	−0.04
				(0.10)	(0.10)	(0.11)	(0.10)	(0.10)	(0.11)
									
Constant	0.09	0.02	0.58****	−0.02	−0.14*	0.19**	−0.03	−0.15	0.69****
	(0.10)	(0.10)	(0.09)	(0.08)	(0.08)	(0.09)	(0.10)	(0.12)	(0.11)
*N*	1,038	1,038	880	1,034	1,034	876	1,034	1,034	876
R-squared	0.03	0.01	0.05	0.03	0.02	0.01	0.03	0.03	0.05

The *ρ* parameter of purely selfless (*α* = 0) and purely selfish (*α* = 1) subjects, who always give nothing or everything, cannot be identified. In the regressions reported in columns 3, 6, and 9, we thus omit purely selfless and purely selfish using a one-sided test at the 10% level. SEs are in parenthesis, bootstrapped using 500 repetitions. *P < 0.10, **P < 0.05, ***P < 0.01, and ****P < 0.001.

Most importantly, in the general population, we find no relationship between the estimated *α* parameters and age (column 2) and a positive relationship between the estimated *α* parameters and income (column 5); the latter relationship may reflect higher incomes among (money-motivated) selfish individuals or an increased selfishness that results from higher income. Overall, these results suggest that the distinctive altruism—the distinctively low *α* estimates—of physicians appears to be linked to the practice of medicine as a “treatment” to social preferences (and indeed the physician–student comparison may understate this effect, given the association between selfishness and wealth, since physicians’ higher incomes on their own may be expected to be associated with greater selfishness).^##^ On the other hand, there is a strong negative relationship between the estimated *ρ* parameters and age (column 3) as well as a negative but less strong relationship to income (column 6). These results indicate that the gap in equality–efficiency orientation between physicians and medical students does not necessarily result from medical practice but rather may reflect the general shift toward equality–orientation that comes with aging; this interpretation is consistent with the earlier finding that physicians’ equality–efficiency orientation is similar to those of the general population. Turning finally to CCEI scores, we find no correlation with age (column 1), but a positive correlation with (contemporaneous) income (column 4); this would at least superficially suggest that part of the higher economic rationality among physicians relative to medical students could reflect their higher incomes, rather than their experience practicing medicine. The relationships are almost identical when we control for both age and income simultaneously (columns 7 through 9).

We especially emphasize our finding that practicing physicians are more altruistic than medical students, because it runs counter to commonly stated assumptions that a “hidden curriculum”—resulting from increasing exposure to practicing physicians as a student progresses from the first to the fourth year of medical school—reduces students’ altruism between when they enroll in medical school and when they graduate (34). While we do not have an explanation for this counterintuitive finding, we note that it is potentially consistent with that in Attema et al. (35), who found that medical students’ altruism decreased during preclinical and clinical studies and increased after they were exposed to medical practice. We consider two possible explanations beyond increased altruism from exposure to the practice of medicine.

– First, the higher level of altruism among physician subjects compared with medical students may reflect selection bias due to the ways in which physician subjects were recruited. As reported in *Overview*, we do not find significant differences by method of recruitment, although the relatively large SEs prevent us from drawing definitive conclusions. (*SI Appendix*, Table A2, column 4).
– Second, the altruism differences between physicians and medical students may reflect generational differences specific to the medical profession, such as changes in how medical schools select candidates over time or changes in the type of individuals drawn to the field of medicine. While we cannot rule out this possibility, we note that we do not observe differences in altruism by age within physician subjects (*SI Appendix*, Table A2, columns 3 and 4).

## Discussion

In this paper we document the distinctive altruism of physicians. Physician subjects are more altruistic than samples drawn from the US population, from a sample of elites, and from students at US medical schools. By contrast, physicians’ equality–efficiency orientations are indistinguishable from those of the general population.

Of particular relevance to the current study is the small subset of papers that focus specifically on physicians’ social preferences. Galizzi et al. (36) summarize the main theoretical and empirical work in the health economics literature on the social preferences of physicians (and other health-care workers). In contrast to our approach, this literature focuses almost exclusively on altruism (i.e., there is no consideration of the equality–efficiency trade-offs that we also capture in our experiment), where altruism is modeled as the relative utility weight placed on the patient’s health benefits versus physicians’ own monetary payoffs.

Following the seminal papers of Ellis and McGuire (31, 37) and Ma and McGuire (38), a number of theoretical studies have incorporated social preferences in physicians’ utility functions—including Jack (39), Choné and Ma (40), and Liu and Ma (41), among others. Despite this abundance of theoretical research, empirical exploration of social preferences among physicians remains quite limited. Prior research has studied altruism (but not equality–efficiency orientation) in medical students and, to a much more limited extent, in physicians, using surveys (4), incentivized discrete choice experiments (42–45), and observational data (46, 47). This work, while important, also leaves important questions unanswered.

In particular, studies using observational (nonexperimental) data to examine physicians’ internalization of patient benefit (or cost) may not involve trade-offs against physicians’ own benefit, such as in medication prescribing (46) and physician assessment of patient long-term care needs (47), and are therefore inconclusive on how physicians trade-off own versus patient interests. Conversely, studies that examine physician-response to financial incentives (such as changes in payment rates) often find that such responses had little impact on patient health (48–50), again making it difficult to evaluate whether physicians intentionally sacrifice patient benefit for their own profit. The findings we report above sidestep such concerns by measuring social preferences in a controlled laboratory setting that, by construction, is free of confounds such as the productivity of medical expenditures.

We thus contribute most directly to the literature that employs economic experiments to study social preferences using a medical framing and dictator-style discrete choice experiments in which the subject chooses among varying levels of service intensity that correspond to different profits for the subject and health benefits for the patients (42, 44, 45, 51, 52). Yet, in contrast to our sample population, subjects in these studies are generally medical students or others, rather than practicing physicians. A prominent exception is Wang et al. (42), who experimentally measured the social preferences of physicians as compared with those of Chinese and German medical students. They found generally similar preferences across samples. However, that study included only 99 physicians working in only one setting: community health service centers in China. To the best of our knowledge, only one study (43) examines efficiency orientation in a medical context; it finds that medical students exhibit concerns for efficiency when choosing among treatment decisions in a laboratory experiment. The paper does not, however, explicitly measure the trade-off between equality and efficiency.

Taking stock of the body of evidence to date, we see our study as making several advances relative to prior work. First, to our knowledge ours is the only study that includes a relatively large number of physicians from different specialties and types of practices. Second, our experimental methodology has been validated based on subject behavior and decisions outside of the laboratory in a variety of settings and populations, whereas evidence of external validity is largely lacking in other studies. Third, ours is the only study that empirically examines equality–efficiency orientation among practicing physicians. Finally, it is the only study that compares physicians’ altruism and equality–efficiency orientation with those of the general population, an “elite” subsample of the population, and a nationwide sample of medical students, which provide important benchmarks.

Our results should be interpreted with caution. Although external validity has been established for our experimental method in other settings and populations, it has not been established in the medical practice of physicians. It is possible that physicians who are more altruistic in our experiment may not behave altruistically—or may behave even more altruistically—when actually taking care of patients. In an experimental setting, there is abundant evidence that absolute altruism is influenced by framing; in the real world, people can also exhibit varying level of altruism across different circumstances (such as winning a game versus donating to a charity). Experimental results concerning absolute altruism may thus not translate directly to any particular setting outside of the laboratory. However, our comparative results about relative altruism across subgroups are robust and are promising in predicting relative preferences and differences in behavior outside of the laboratory across subgroups.

The above limitations notwithstanding, the fact that most physicians did not behave altruistically in our study and that they did not display a stronger preference for efficiency than other samples suggest that policymakers should not depend on physician professionalism alone in ensuring high-quality medical care or the efficient use of medical resources. In fact, policymakers are increasingly using bureaucratic mechanisms to monitor and constrain physician behavior and using financial incentives to influence physicians’ decisions.

However, our finding that physicians are on average more altruistic than others suggests that physician professionalism is not merely a self-serving myth. Rather, it suggests that many physicians will likely put patients first in the many important areas of care in which their performance is not rewarded financially and likely cannot be measured. While health-care policy may need conventional incentives such as pay for performance to promote good behavior from physicians who are self-interested, the material share of physicians who are altruistic suggests that policymakers should also consider whether specific bureaucratic mechanisms and financial incentives might have the unintended consequence of reducing physician altruism (53, 54), either by directly changing their preferences or by selecting less altruistic individuals into the profession, and consider altering or abandoning policies that seem likely to have these effects.

## Materials and Methods

### GARP.

The preference ordering of self ≿ can be represented by a utility function $u_{s}\left(\pi_{s},\pi_{o}\right)$ that captures the possibility of giving if $u_{s}\left(\pi_{s},\pi_{o}\right)\geq u_{s}\left(\pi \prime_{s},\pi \prime_{o}\right)$ whenever $\left(\pi_{s},\pi_{o}\right)≿\left(\pi^{\prime }{}_{s},\pi^{\prime }{}_{o}\right).$ Afriat’s(23) theorem tells us that if the data satisfy GARP then there exists an underlying utility function $u_{s}\left(\pi_{s},\pi_{o}\right)$ that rationalizes the data and that $u_{s}\left(\pi_{s},\pi_{o}\right)\;$ can be chosen to be increasing, continuous and concave. In the case of two goods, consistency (completeness and transitivity) and budget balancedness imply that demand functions must be homogeneous of degree zero. Assuming also separability and homotheticity, the underlying utility function $u_{s}\left(\pi_{s},\pi_{o}\right)$ must be a member of the CES family.

### CCEI.

See Fisman et al. (16) for details on how we calculate subjects’ CCEI scores and a discussion of various alternative measures that have been proposed for this purpose. Most importantly, Fisman et al. (16) also show that if utility maximization is not in fact the correct model, then our experiment is sufficiently powerful to detect this. We follow Bronars (55), which builds on Becker (56), and compare the behavior of our actual subjects with the behavior of simulated subjects who randomize uniformly on each budget line. Mean CCEIs for a random sample of 25,000 simulated subjects are only 0.60. As another confirmation, Fisman et al. (16) generated a benchmark level of consistency using hypothetical subjects with an idiosyncratic preference shock that has a logistic distribution. See Chambers and Echenique (57) for a broader discussion of developments in revealed preference theory.

### CES Specification and Estimation.

The CES expenditure function of tokens to self $p_{s}\pi_{s}$ is given by

$$p_{s}\pi_{s}=\frac{g}{{\left(p_{s}/p_{o}\right)}^{r}+g},$$

where r=ρ/(1−ρ) and $g={\left[\alpha /(1-\alpha )\right]}^{1/(1-\rho )},$ which is bounded between 0 and 1, as the endowment is normalized to 1. Note that if ρ>0 (resp. ρ<0) then r>0 (resp. r<0) so an increase in the relative price of allocating tokens to self, $p_{s}/p_{o}$, lowers (resp. raises) the expenditure share of the tokens allocated to self $p_{s}\pi_{s}.$ When ρ→0 (so r→0), the CES form approaches Cobb–Douglas $\pi_{s}^{\alpha }\pi_{o}^{1-\alpha }$ and expenditures on tokens allocated to self $p_{s}\pi_{s}$ is invariant to the price ratio $p_{s}/p_{o}$ and equal to α.

We generate estimates of g and r  using nonlinear tobit maximum likelihood and use these estimates to infer the values of the underlying CES parameters *α* and *ρ*. We emphasize again that the graphical representation enables us to collect 50 observations per subject and therefore that our estimations can be done for each subject separately. This allows us to capture the heterogeneity of social preferences. We refer the interested reader to ref. 18 for more details on the individual-level estimation of *α* and *ρ*.

## Supplementary Material

Supplementary File

## Data Availability

The data and code that support the findings of this study are available publicly at https://drive.google.com/drive/folders/1iwNwacZ6Tkqnp-AJJxoZMePHYGEAUAJb?usp=sharing (58).

## Footnotes

^*^For example, requiring prior authorization from the insurer for expensive services (bureaucratic mechanism) and rewarding medical groups that maintain quality of care while having relatively low overall costs of caring for patients (financial incentives).

^†^Put differently, we present subjects with a decision problem that can be interpreted as a standard economic problem—the selection of a bundle from a standard budget set. These decision problems are presented using a graphical experimental interface that allows for the collection of a rich individual-level dataset. Additionally, our experiments employ decision problems that are representative (both in the statistical sense and in the economic sense) of broad classes of other-regrading choices rather than being narrowly tailored to capture a particular behavior in a specific context.

^‡^In addition, this prior work shows that our experimentally generated estimates of social preferences are strongly correlated, in intuitive ways, with behaviors outside the laboratory in natural decision environments, providing evidence for external validity of the experimental approach. We provide further details on this point below.

^§^The assumption of rational choice in economics “simply” requires consistent preferences over all possible alternatives and choices that correspond to the most preferred alternative from the feasible set. Insofar as social preferences are rational, then the techniques of economic analysis may be brought to bear on modeling and predicting behavior governed by these preferences.

^¶^The ALP is a longitudinal survey administered online by the RAND Corporation. The sample consists of more than 5,000 individuals recruited from a broad cross-section of the US population. Via the internet, the ALP can implement sophisticated experiments and collects a great deal of individual demographic and economic information from its participants. See the ALP website (https://www.rand.org/research/data/alp.html) for detailed information.

^#^In the experiment with physicians, other was an anonymous respondent in the UAS. Like the ALP, the UAS is a broadly representative panel of approximately 6,000 adult respondents, one that is administered by the University of Southern California. The UAS, which began in 2014, expanded on the ALP which began in 2006. See the UAS website (https://uasdata.usc.edu/index.php) for more information. In the experiments with ALP respondents and medical students, other was an anonymous ALP respondent (not sampled for the experiment).

^ǁ^The power of the experiment depends on two factors. The first is that the number of decisions made by each subject is large. This is a crucial point, because in most experimental studies, the number of individual decisions is too small to provide a powerful test. The second is that the range of choice sets is generated so that budget lines cross frequently.

^**^The average annual income of internists ($230,000) and cardiologists ($430,000) correspond to hourly earnings of approximately $120 and $225, respectively, while the ALP subjects’ average annual income of $55,000 is approximately $30 per h; medical residents’ hourly wages are similarly just under $30. All calculations assume a 40-h work week multiplied by 50 wk.

^††^Bootstrapped Kolmogorov–Smirnov tests (1,000 repetitions) reject the equality of the CDF of the CCEI scores of the physician sample and ALP, ALP elites, and medical students—*P* values less than 0.001.

^‡‡^The equality–efficiency orientation (ρ) of purely selfish (α=1) subjects and purely selfless (α=0), who always give nothing or everything to other, cannot be identified, because their choices are invariant to the price of giving. We use t tests to classify individual types and omit those for whom we cannot reject that α=1 or α=0 (using a one-sided test at 10% significance) from the CDFs in Fig. 2*B* because the estimates of ρ are quite noisy for these subjects.

^§§^Bootstrapped Kolmogorov–Smirnov tests (1,000 repetitions) reject the equality of the CDF of the estimated altruism parameter (α) of the physician sample and ALP (P<0.001), ALP elites (P=0.094), and medical students (P<0.001). The corresponding *P* values for the bootstrapped Kolmogorov–Smirnov tests of the CDF of the estimated equality–efficiency orientation (ρ) parameter are 0.013, 0.358, and <0.001, respectively. The *P* values for the bootstrapped Kolmogorov–Smirnov tests of the CDF of the estimated equality–efficiency orientation (ρ) parameter for ALP, ALP elites, and medical students are 0.013, 0.358, and <0.001, respectively. The statistically significant difference in the CDF between physicians and general ALP could be due to differences in the location and shape of the two (unadjusted) distributions and the relatively large sample size of both, relative to the ALP elite sample. The two CDFs nonetheless cross over each other.

^¶¶^In *SI Appendix*, Table A1 we present tobit specifications with the CCEI and the CES altruism parameter (*α*) as the dependent variables. The tobit specifications adjust for censoring of the dependent variable at one. Since the distribution of the CES equality-efficient orientation parameter (*ρ*) is skewed, we estimate a median regression that is less sensitive to extreme values.

^##^The average income of our physician sample is likely much higher than even the top quintile of the ALP sample or the ALP elite sample (both about $125,000). There was no significant difference in α in the subset of the general ALP sample with incomes above $200,000 compared with those with incomes between $100,000 and $200,000 (both have an average *α* of about 0.70, as compared with 0.61 for the physician sample). The same holds true for the ALP elite sample.
