# Supplementary material for: Experimental evidence of physician social preferences
Source: Proc Natl Acad Sci U S A. 2022 Jul 6;119(28):e2112726119. doi: 10.1073/pnas.2112726119 (PMC9282368; doi:10.1073/pnas.2112726119)
Supplement: Supplementary File [file pnas.2112726119.sapp.pdf]

Table A1. **Rationality (CCEI), altruism (α) and equality-efficiency orientation (ρ) in the physician sample and the three other samples.**

	CCEI			α			ρ		
	(1) vs. General ALP	(2) vs. ALP elites	(3) vs. MS	(4) vs. General ALP	(5) vs. ALP elites	(6) vs. MS	(7) vs. General ALP	(8) vs. ALP elites	(9) vs. MS
Physicians	0.14****	0.10****	0.04****	-0.07****	-0.09***	-0.11****	-0.01	-0.12	-0.53****
	(0.01)	(0.02)	(0.01)	(0.02)	(0.03)	(0.02)	(0.09)	(0.26)	(0.10)
Female	Yes	Yes	Yes	Yes	Yes	Yes	Yes	Yes	Yes
Age	Yes	Yes	No	Yes	Yes	No	Yes	Yes	No
Census region	Yes	Yes	Yes	Yes	Yes	Yes	Yes	Yes	Yes
Mean of	0.88	0.95	0.94	0.66	0.63	0.68	-2.95	-3.70	-2.25
N	1,277	366	787	1,277	366	787	1,069	294	588

Notes: Coefficients shown are from tobit regressions with CCEI (columns 1-3) and α (columns 4-6) as the dependent variable, and median regressions with ρ (columns 7-9) as the dependent variable. The ρ parameter of purely selfless ($\alpha = 0$) and purely selfish ($\alpha = 1$) subjects, who always give nothing or everything to other, cannot be identified. In the regressions reported in columns 7-9, we thus omit purely selfless and purely selfish using a one-side t-test at the 10% level. Standard errors are in parenthesis. *, **, ***, and **** stand for 0.10, 0.05, 0.01, and 0.001 significance level, respectively.

Table A2. **Differences in rationality (CCEI), altruism (α) and equality-efficiency orientation (ρ) among physicians.**

	CCEI		α		ρ	
	(1)	(2)	(3)	(4)	(5)	(6)
Specialty (omitted: Primary Care)						
Cardiology	-0.15	-0.14	-0.03	-0.16	-0.04	-0.06
	(0.16)	(0.19)	(0.15)	(0.20)	(0.16)	(0.21)
Practice type (omitted: Hospital)						
Private	0.06	0.10	-0.17	-0.24	0.07	-0.09
	(0.16)	(0.18)	(0.19)	(0.20)	(0.20)	(0.23)
Cardiology × Private	0.02	-0.02	0.26	0.40	-0.09	-0.01
	(0.32)	(0.35)	(0.30)	(0.34)	(0.28)	(0.35)
Female	-0.02	-0.02	-0.18	-0.20	-0.08	-0.11
	(0.12)	(0.12)	(0.13)	(0.13)	(0.14)	(0.15)
Age (omitted: ≤39)						
40-49	0.24	0.25*	-0.04	-0.05	-0.17	-0.17
	(0.15)	(0.15)	(0.15)	(0.16)	(0.16)	(0.17)
50-59	0.03	0.05	0.02	-0.01	-0.25	-0.25
	(0.17)	(0.17)	(0.18)	(0.18)	(0.20)	(0.20)
≥ 60	-0.13	-0.17	-0.17	-0.19	-0.28	-0.27
	(0.20)	(0.19)	(0.19)	(0.18)	(0.21)	(0.20)
Practice size (number of physicians) (omitted: ≤ 35)						
36-100		-0.28		0.13		-0.04
		(0.18)		(0.20)		(0.22)
101-350		-0.61**		0.30		0.16
		(0.25)		(0.23)		(0.26)
350-1600		-0.19		0.18		-0.09
		(0.23)		(0.27)		(0.28)
Region of practice (omitted: Northeast)						
Midwest		0.05		-0.08		-0.36
		(0.21)		(0.21)		(0.23)
South		0.02		-0.02		-0.02
		(0.19)		(0.23)		(0.24)
West		-0.07		-0.48***		-0.22
		(0.20)		(0.17)		(0.22)
Method of recruitment (omitted: Author's contact)						
Referred by		-0.22		-0.07		-0.07
author's contact		(0.15)		(0.15)		(0.18)
Web search		-0.32		0.41		0.24
		(0.34)		(0.31)		(0.40)
Constant	-0.03	0.39*	0.14	0.19	0.19	0.42
	(0.13)	(0.20)	(0.13)	(0.23)	(0.15)	(0.29)
N	284	284	284	284	231	231
R squared	0.02	0.07	0.01	0.05	0.01	0.04

Notes: Rank regressions transforming the parameters CCEI (columns 1-2), α (columns 3-4), and ρ (columns 5-6) from the physician sample into an overall ranking, which is then normalized to have mean zero and standard deviation (SD) one. The ρ parameter of purely selfless ($\alpha = 0$) and purely selfish ($\alpha = 1$) subjects, who always give nothing or everything to other, cannot be identified. In the regressions reported in columns 5-6, we thus omit purely selfless and purely selfish using a one-side test at the 10% level. Standard errors are in parenthesis, bootstrapped using 500 repetitions. *, **, ***, and **** stand for 0.10, 0.05, 0.01, and 0.001 significance level, respectively.

Table A3: **The effect of age and income on rationally (CCEI), altruism (α) and equality-efficiency orientation (ρ) in the ALP samples.**

	(1) CCEI	(2) α	(3) ρ	(4) CCEI	(5) α	(6) ρ	(7) CCEI	(8) α	(9) ρ
Female	-0.26****	-0.14	-0.14	-0.24****	-0.11	-0.12	-0.24****	-0.10	-0.15
	(0.07)	(0.09)	(0.12)	(0.07)	(0.09)	(0.11)	(0.07)	(0.09)	(0.11)
Elite	0.42***	0.20	0.11						
	(0.14)	(0.17)	(0.22)						
Age									
31-39	-0.02	0.02	-0.59***				-0.02	0.01	-0.59***
	(0.12)	(0.17)	(0.20)				(0.12)	(0.17)	(0.19)
40-49	0.07	-0.04	-0.63***				0.05	-0.04	-0.56***
	(0.12)	(0.17)	(0.20)				(0.12)	(0.17)	(0.19)
50-59	0.13	0.05	-0.59***				0.10	0.02	-0.51***
	(0.11)	(0.15)	(0.19)				(0.11)	(0.15)	(0.18)
$\geq$ 60	0.03	0.31**	-0.89****				-0.00	0.27*	-0.88****
	(0.11)	(0.15)	(0.19)				(0.11)	(0.15)	(0.18)
Income									
2nd Quintile				0.14	0.22	-0.27	0.15	0.20	-0.26
				(0.10)	(0.15)	(0.17)	(0.10)	(0.15)	(0.16)
3rd Quintile				0.13	0.40***	-0.35**	0.14	0.36***	-0.27*
				(0.10)	(0.13)	(0.16)	(0.10)	(0.13)	(0.15)
4th Quintile				0.26**	0.45***	-0.57****	0.25**	0.44***	-0.55****
				(0.11)	(0.14)	(0.17)	(0.11)	(0.14)	(0.16)
5th Quintile				0.38****	0.42***	-0.13	0.38****	0.43***	-0.12
				(0.11)	(0.15)	(0.18)	(0.11)	(0.15)	(0.17)
Constant	0.13	-0.02	0.69****	0.03	-0.22*	0.36***	-0.00	-0.28	0.90****
	(0.10)	(0.15)	(0.18)	(0.09)	(0.12)	(0.14)	(0.12)	(0.18)	(0.19)
N	1,038	1,038	880	1,034	1,034	876	1,034	1,034	876

Notes: The association between age (columns 1-3, 7-9) and income (columns 4-6, 7-9) and our measures of rationality and social preferences – CCEI (columns 1, 4 and 7), α (columns 2, 5 and 8), and ρ (columns 3, 6 and 9). The ρ parameter of purely selfless ($\alpha = 0$) and purely selfish ($\alpha = 1$) subjects, who always give nothing or everything to other, cannot be identified. In the regressions reported in columns 3, 6 and 9, we thus omit purely selfless and purely selfish using a one-side test at the 10% level. Robist standard errors are in parenthesis.*, **, ***, and **** stand for 0.10, 0.05, 0.01, and 0.001 significance level, respectively.
